# A local internal lateral fixation (LILF) of dura mater (DM) to the wall of the vertebral channel as the main reason of the serious idiopathic scoliosis (IS). How to see it with the MRI test, and how to forecast early the scoliosis worsening (bad scenario). How to stop this scoliosis

**DOI:** 10.1186/1748-7161-8-S1-P14

**Published:** 2013-06-03

**Authors:** VV Drobyshevskij

**Affiliations:** 1 The S&R Research Institute for Child Orthopaedics, Saint-Petersburg, Russia

## Introduction

There are post-mortem investigations of serious scoliosis with a LILF of the DM. In early research, it was considered to be a secondary phenomenon, as a consequence of the vertebra’s edges pressure in the vertebral channel, towards the dura mater.(Movshovich,1964).

## Aims

First, to prove that the LILF of the DM is not the secondary phenomenon. On the contrary, the LILF of the DM, like a hooked bowstring, causes a serious idiopathic scoliosis. Second, to create an MRI test, so any competent scoliosis expert, could see an initial LILF of the DM, and predict early a dangerous development of scoliosis.

## Methods

We used the Cheneau - Abbott type braces with the non-magnetic parts, and the side position of patient for MRI-test. We investigated the form, and the locations, of the spinal cord in the corrected position of the scoliosis spine.

## Results

We find that the starting scoliosis has LILF of DM in several cases. These are serious scoliosis in future.

## Conclusions

The MRI test, for a LILF of the DM, can help to make a forecast of the future development of scoliosis. We can make the early scoliosis treatment easy, by separation of the LILF of the DM, like Edville Gerhardt Abbott (1913), by overcorrection brace, or maybe by micro invasive surgical releasing in the future.
